# Supplementary material for: Designing a deposit-refund system for cigarette butts: What do smokers care about?
Source: PLoS One. 2025 Oct 22;20(10):e0335205. doi: 10.1371/journal.pone.0335205 (PMC12543133; doi:10.1371/journal.pone.0335205)
Supplement: S1 Appendix — (DOCX) [file pone.0335205.s001.docx]

| 1 | The more I know about the impact that cigarette butts cause, the more things I feel guilty about. |
| --- | --- |
| 2 | I am constantly angry with myself because I think that I am not doing enough and that I am harming the environment by my very existence. |
| 3 | It makes me feel uneasy that I am part of a system that is amplifying cigarette butts. |
| 4 | I feel guilty for not paying enough attention to the issue of cigarette butts. |
| 5 | At times I feel some personal responsibility for the problems and unfolding impacts of cigarette butts. |
