# Supplementary material for: Designing a deposit-refund system for cigarette butts: What do smokers care about?
Source: PLoS One. 2025 Oct 22;20(10):e0335205. doi: 10.1371/journal.pone.0335205 (PMC12543133; doi:10.1371/journal.pone.0335205)
Supplement: S2 Appendix — * Indonesia only differs in the assumption that one pack of cigarettes (16 sticks) is priced at IDR 32,000 or IDR 2,000 per stick. (DOCX) [file pone.0335205.s002.docx]

| Now, please imagine a mandatory deposit-refund system for cigarette butts. Under this system, you must pay an additional fee when purchasing cigarettes. This fee is refundable (fully or partially, depending on the assumed refund rate) when you return cigarette butts to designated return points. Please assume that one pack of cigarettes (20 sticks) is priced at 500 JPY or 25 JPY per stick. |
| --- |
| 1. The collected cigarette butts will undergo environmentally friendly disposal processes to mitigate the hazardous risks associated with improper disposal. It includes recycling (e.g., for use in making bricks for construction, generating energy, and producing insecticides). |
| 1. You can return cigarette butts at any number you desire. |
|  |
| In each of the following questions, you will be introduced to two systems with different deposit, refund rate, management institution, and accessibility. |
| 1. Deposit: An additional fee paid when purchasing cigarettes that is refundable (fully or partially depending on the assumed refund rate) upon your returning cigarette butts. |
| 1. Refund rate: Refundable deposit that you can receive upon your returning cigarette butts. For example, when the deposit is 100 JYP/pack, 50% refund rate means that you receive 50 JPY/pack. The remaining amount is complemented for operating the deposit-refund system under the assumed management institution. |
| 1. Management Institution: The organization that is responsible for operating the deposit-refund system. |
| 1. Accessibility: Time you need to reach the designated return points. |
