# Supplementary material for: Designing a deposit-refund system for cigarette butts: What do smokers care about?
Source: PLoS One. 2025 Oct 22;20(10):e0335205. doi: 10.1371/journal.pone.0335205 (PMC12543133; doi:10.1371/journal.pone.0335205)
Supplement: S3 Appendix — (DOCX) [file pone.0335205.s003.docx]

|  | Japan (n = 1,865) | | Indonesia (n = 2,000) | |
| --- | --- | --- | --- | --- |
|  | CL 1 | CL 2 | CL 1 | CL 2 |
|  | Control  (n = 911) | Treatment  (n = 954) | Control  (n = 1,000) | Treatment  (n = 1,000) |
| Mean coefficients |  |  |  |  |
| ASC | -0.364*** | 0.030 | 1.512*** | 1.762*** |
|  | (0.085) | (0.082) | (0.089) | (0.092) |
| Deposit | 0.000 | 0.000 | 0.000*** | 0.000*** |
|  | (0.000) | (0.000) | (0.000) | (0.000) |
| Refund rate | 0.741*** | 0.509*** | 0.498*** | 0.503*** |
|  | (0.090) | (0.087) | (0.088) | (0.089) |
| Management institution | 0.050* | 0.085*** | 0.059** | 0.076*** |
|  | (0.021) | (0.020) | (0.019) | (0.019) |
| Accessibility | -0.022*** | -0.020*** | -0.015*** | -0.021*** |
|  | (0.002) | (0.002) | (0.002) | (0.002) |
| Opt-out frequencies | 0.3608 | 0.3017 | 0.0967 | 0.0818 |
| Adjusted R² | 0.015 | 0.013 | 0.147 | 0.172 |
| AIC | 11831.61 | 12416.88 | 11239.46 | 10914.72 |
| BIC | 11864.65 | 12450.14 | 11272.96 | 10948.22 |
| Log-likelihood | -5910.807 | -6203.438 | -5614.729 | -5452.361 |
| Num. events | 5466 | 5724 | 6000 | 6000 |
