# Supplementary material for: Designing a deposit-refund system for cigarette butts: What do smokers care about?
Source: PLoS One. 2025 Oct 22;20(10):e0335205. doi: 10.1371/journal.pone.0335205 (PMC12543133; doi:10.1371/journal.pone.0335205)
Supplement: S4 Appendix — (DOCX) [file pone.0335205.s004.docx]

|  | Variables | df | t | *p-*values |
| --- | --- | --- | --- | --- |
| 1 | Cigarettes consumption | 1928.7 | 3.37 | < .001 |
| 2 | Littering frequency | 3861.7 | -29.18 | < .001 |
| 3 | Littering frequency  (inferred valuation) | 3359.6 | -44.10 | < .001 |
| 4 | Eco-guilt | 3257.4 | -34.23 | < .001 |
