# Supplementary material for: Designing a deposit-refund system for cigarette butts: What do smokers care about?
Source: PLoS One. 2025 Oct 22;20(10):e0335205. doi: 10.1371/journal.pone.0335205 (PMC12543133; doi:10.1371/journal.pone.0335205)
Supplement: S5 Appendix — We adopted Krinsky and Robb’s method to calculate 95% confidence intervals [48]. The WTP for accessibility appears different in Fig 7 because it has been multiplied to reflect the respective attribute levels. (DOCX) [file pone.0335205.s005.docx]

| WTP management institution | |  |  |  |
| --- | --- | --- | --- | --- |
|  | Japan | | Indonesia | |
|  | Control (n = 911) | Treatment (n = 954) | Control (n = 1,000) | Treatment (n = 1,000) |
| Mean | 16.79 | 47.19 | 404.67 | 631.90 |
| 2.5% quantiles | 6.59 | 25.43 | 135.99 | 325.00 |
| 97.5% quantiles | 29.84 | 102.94 | 710.43 | 1029.30 |
| Standard error | 5.93 | 19.77 | 146.54 | 179.67 |
|  |  |  |  |  |
| WTP accessibility |  |  |  |  |
|  | Japan | | Indonesia | |
|  | Control (n = 911) | Treatment (n = 954) | Control (n = 1,000) | Treatment (n = 1,000) |
| Mean | -5.48 | -9.38 | -84.42 | -151.90 |
| 2.5% quantiles | -8.21 | -20.48 | -126.00 | -222.20 |
| 97.5% quantiles | -3.90 | -5.83 | -53.26 | -108.80 |
| Standard error | 1.10 | 3.74 | 18.56 | 28.93 |
