# Supplementary material for: Designing a deposit-refund system for cigarette butts: What do smokers care about?
Source: PLoS One. 2025 Oct 22;20(10):e0335205. doi: 10.1371/journal.pone.0335205 (PMC12543133; doi:10.1371/journal.pone.0335205)
Supplement: S6 Appendix — (DOCX) [file pone.0335205.s006.docx]

|  | Variables | df | t | *p-*values |
| --- | --- | --- | --- | --- |
| 1 | Japan control CBs vs. HNB | 1261.7 | -12.68 | < .001 |
| 2 | Japan treatment CBs vs. HNB | 1296.2 | -16.33 | < .001 |
| 3 | Indonesia control CBs vs. HNB | 1021.4 | 5.60 | < .001 |
| 4 | Indonesia treatment CBs vs. HNB | 1013.9 | 0.89 | 0.19 |
| 5 | Japan control HNB vs. Indonesia control HNB | 1139.5 | 3.93 | < .001 |
| 6 | Japan treatment HNB vs. Indonesia treatment HNB | 1106.7 | 3.55 | < .001 |
