# Supplementary material for: Designing a deposit-refund system for cigarette butts: What do smokers care about?
Source: PLoS One. 2025 Oct 22;20(10):e0335205. doi: 10.1371/journal.pone.0335205 (PMC12543133; doi:10.1371/journal.pone.0335205)
Supplement: S2 Supplementary — (DOCX) [file pone.0335205.s008.docx]

Supplementary material

Sample questionnaire

| Note to readers:  Among the eight surveys conducted in our study, this questionnaire was specifically targeted at the Japanese treatment group, BLOCK 1, who received environmental information and completed a comprehension test based on that information. Except for the choice sets, the other treatment group (BLOCK 2) answered the same questions. The control questionnaire was identical to the treatment questionnaires, except it did not include the environmental information or the comprehension test. |
| --- |

**Sociodemographic**

1. What is your age? For example: 20.

_______________ years

1. What is your gender?
2. Male
3. Female
4. Please select your annual household income.
5. Below 1,000,000 JPY
6. 1,000,000 to below 2,000,000 JPY
7. 2,000,000 to below 3,000,000 JPY
8. 3,000,000 to below 4,000,000 JPY
9. 4,000,000 to below 5,000,000 JPY
10. 5,000,000 to below 6,000,000 JPY
11. 6,000,000 to below 7,000,000 JPY
12. 7,000,000 to below 8,000,000 JPY
13. 8,000,000 to below 9,000,000 JPY
14. 9,000,000 to below 10,000,000 JPY
15. 10,000,000 to below 12,000,000 JPY
16. 12,000,000 to below 15,000,000 JPY
17. 15,000,000 to below 18,000,000 JPY
18. 18,000,000 to below 20,000,000 JPY
19. Above 20,000,000 JPY
20. Please select the highest level of education you have attained.
21. Junior high school
22. High school
23. Vocational school
24. College
25. Graduate school

**Behavior**

1. In a day, how many cigarettes do you smoke? For example: 1

_______________ cigarettes

1. How often do you litter cigarette butts on the ground or in a sewer drain?
2. Never
3. Rarely
4. Sometimes
5. Often
6. Always
7. In your opinion, how often do other smokers litter cigarette butts on the ground or in a sewer drain?
8. Never
9. Rarely
10. Sometimes
11. Often
12. Always

**Attitude**

1. Please read the following statements and select the option that best matches your opinion.

|  | Statements | Strongly disagree | Disagree | Neither agree nor disagree | Agree | Strongly agree |
| --- | --- | --- | --- | --- | --- | --- |
| 1 | The more I know about the impact that cigarette butts cause, the more things I feel guilty about. |  |  |  |  |  |
| 2 | I am constantly angry with myself because I think that I am not doing enough and that I am harming the environment by my very existence. |  |  |  |  |  |
| 3 | It makes me feel uneasy that I am part of a system that is amplifying cigarette butts. |  |  |  |  |  |
| 4 | I feel guilty for not paying enough attention to the issue of cigarette butts. |  |  |  |  |  |
| 5 | At times I feel some personal responsibility for the problems and unfolding impacts of cigarette butts. |  |  |  |  |  |

**Awareness**

1. How much do you agree or disagree with the following statements?

| Statements | Strongly disagree | Disagree | Neither agree nor disagree | Agree | Strongly agree |
| --- | --- | --- | --- | --- | --- |
| Cigarette butts are biodegradable |  |  |  |  |  |
| Cigarette butts are toxic to the environment |  |  |  |  |  |
| Cigarette butts are harmless to animals and plants |  |  |  |  |  |
| Cigarette butts are harmless to fish and sea life |  |  |  |  |  |

**Environmental information intervention**

Next, please read the following material carefully. You will be asked to answer a comprehension test to confirm that you fully understand the content.

**
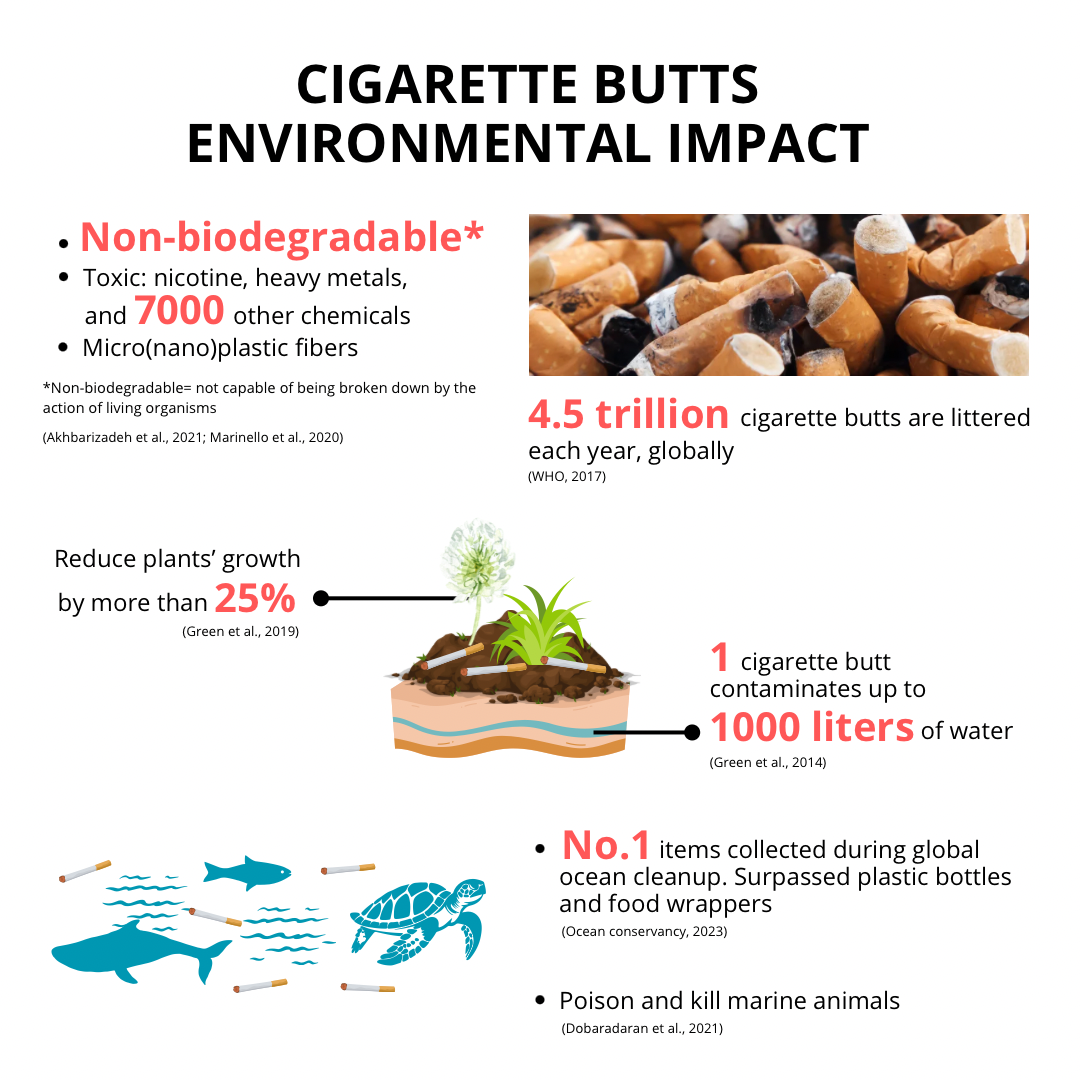
**

1. Based on the content of the material just presented, choose the correct answer.


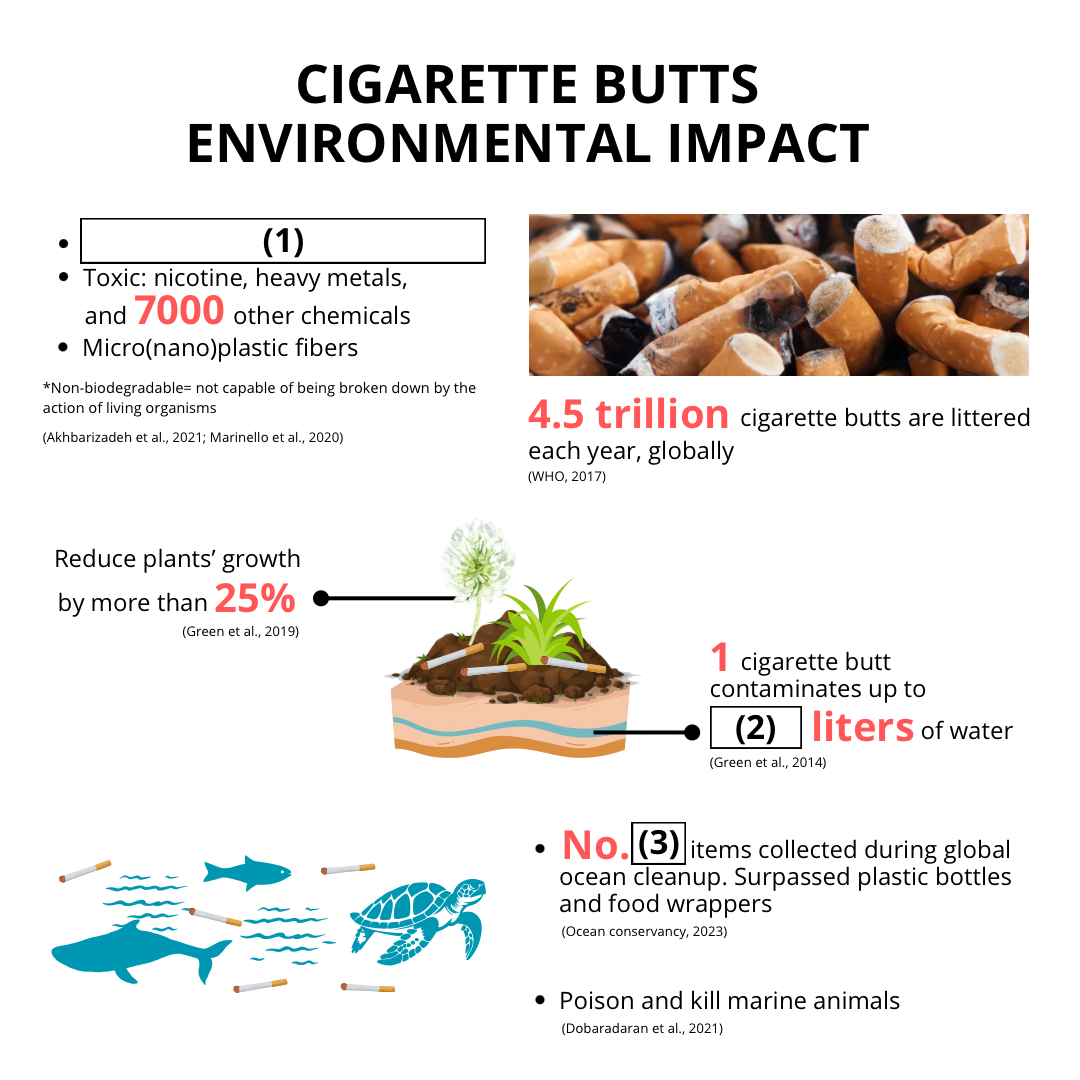


1. (1) Biodegradable, (2) 1000, (3) 1
2. (1) Non-biodegradable, (2) 1, (3) 100
3. (1) Non-biodegradable, (2) 1000, (3) 1
4. (1) Biodegradable, (2) 1, (3) 100

**If fail**

Your selected answer is incorrect. Please review the figure and explanation provided and answer the question again.

| Note to readers:  In the actual surveys, the environmental information and comprehension test were presented in the respective country’s language, in this case Japanese. Please refer to the following:  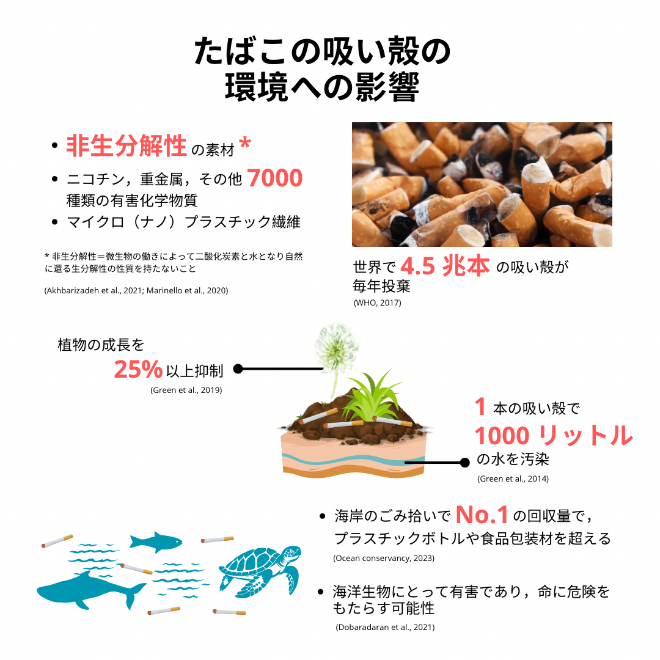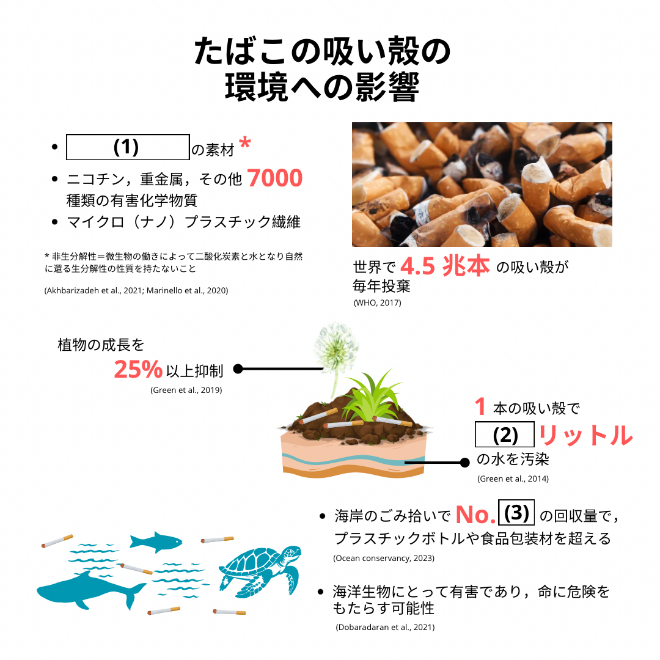 |
| --- |

**DCE choice sets**

Now, please imagine a mandatory deposit-refund system for cigarette butts. Under this system, you must pay an additional fee when purchasing cigarettes. This fee is refundable (fully or partially, depending on the assumed refund rate) when you return cigarette butts to designated places. Please assume that one pack of cigarettes (20 sticks) is priced at 500 JPY or 25 JPY per stick.

1. The collected cigarette butts will undergo environmentally friendly disposal processes to mitigate the hazardous risks associated with improper disposal. It includes recycling (e.g., for use in making bricks for construction, generating energy, and producing insecticides).
2. You can return cigarette butts at any number you desire.

In each of the following questions, you will be introduced to two systems with different deposit, refund rate, management institution, and accessibility.

1. **Deposit**: An additional fee paid when purchasing cigarettes that is refundable (fully or partially depending on the assumed refund rate) upon your returning cigarette butts.
2. **Refund rate**: Refundable deposit that you can receive upon your returning cigarette butts. For example, when the deposit is 100 JYP/pack, 50% refund rate means that you receive 50 JPY/pack. The remaining amount is complemented for operating the deposit-refund system under the assumed management institution.
3. **Management Institution**: The organization that is responsible for operating the deposit-refund system.
4. **Accessibility**: Time you need to reach the designated place to return your cigarette butts for a refund.

[Combination 1]

Please indicate which of the two offered deposit-refund systems you prefer, or whether you prefer neither.

1. I prefer the deposit-refund system 1
2. I prefer the deposit-refund system 2
3. I prefer neither of them

| Note to readers:  Five additional combinations are presented below. For the complete choice set, please refer to Supplementary Material 1. |
| --- |

**Perception toward deposit-refund system**

1. What is your thought, image, or word about deposit-refund system for cigarette butts? Please write your response in full sentences or as single words.

_____________________________________________________________________

**Heat-not-burn cigarettes**

1. Have you ever used heat-not-burn tobacco products? If not, are you familiar with how it works?
2. I have used it.
3. I have not used it, but I know how it works.
4. I have not used it, and I do not know how it works.
5. This survey has asked about your preferences regarding the deposit-refund system for cigarette butts. When comparing this system with the deposit-refund system for heat-not-burn tobacco products (such as heat-not-burn cigarettes and their devices), which do you think is easier to return? Please assume that the deposit amount, refund rate, managing institution, and accessibility are the same.
6. Cigarette butts are easier to return.
7. Heat-not-burn cigarettes are easier to return.
8. There is no difference.
