# Supplementary material for: Designing a deposit-refund system for cigarette butts: What do smokers care about?
Source: PLoS One. 2025 Oct 22;20(10):e0335205. doi: 10.1371/journal.pone.0335205 (PMC12543133; doi:10.1371/journal.pone.0335205)
Supplement: Attachment — Submitted filename: Response to Reviewers.docx [file pone.0335205.s010.docx]

Response to Reviewers

Reviewer #1: This manuscript presents a well-executed discrete choice experiment (DCE) exploring smokers’ preferences for a deposit-refund system (DRS) for cigarette butts in Japan and Indonesia. It contributes to the literature on extended producer responsibility and behavioural environmental policy by incorporating both stated preferences and an information intervention. The paper is well-organised and clearly written, with sound methodology. However, there are several conceptual and interpretive issues that require attention before publication.

Dear Reviewer #1,

Thank you for your thoughtful and constructive suggestions. We have carefully revised the manuscript, incorporating many of your suggestions as well as those from Reviewer #2.

After thorough consideration, there are a few suggestions we were unable to adopt upon considering how we can make our manuscript better contribute to the literature. We respectfully hope you understand our reasoning in this regard.

Additionally, please note that Figures 7 (WTP monetary comparison) and 8 (WTP percentage comparison) have been updated to correct miscalculations present in the earlier version. These revisions do not alter our overall findings. We have also updated figure numbers, appendix numbers, and citation numbering throughout the manuscript.

Best regards,

Authors

Main comments
1. Scope and Framing of Research Questions
The manuscript presents five research questions, but the mapping between questions, methods, and results is not always clear. For instance, the link between RQ2 (perceptions) and the inductive coding of open-ended responses is weaker than the structured linkages for RQ3–RQ5. I recommend that the authors:
• Clarify which analytical components correspond to each research question.
• Structure the results section to more clearly reflect this mapping.

Our response:

Thank you for bringing this to our attention. We have revised the structure of the results section, so that “Perception of DRS” now precedes “Conditional logit model results” for better clarity. The changes are as follows:

[Previous]

3. Results

3.1 Sample description

3.2 Understanding the environmental impact of CBs

3.3 Conditional logit model results

3.4 Perception of DRS

3.5 Heat-not-burn cigarettes

[Revised]

3. Results

3.1 Sample description

3.2 Understanding the environmental impact of CBs

3.3 Perception of DRS

3.4 Conditional logit model results

3.5 Heat-not-burn cigarettes

Furthermore, we have added the research question numbers to the first sentence of each results section to clarify which analytical components correspond to each research question.

[Revised: LINE 297]

Table 3 presents respondents’ understanding of the environmental impact of CBs (RQ1).

[Revised: LINE 304-305]*

Fig 5 illustrates the results of the inductive coding of respondents’ thoughts, ideas, or word associations toward a DRS for CBs (RQ2).

[Revised: LINE 327-328]

Table 4 presents the conditional logit estimates for the control (without environmental information), and the treatment groups (with environmental information) (RQ3, RQ4, RQ5).

[Revised: LINE 385-387]

Accordingly, Japanese respondents showed significantly higher DRS preferences for HNB cigarettes than Indonesian respondents in both the control and treatment groups (Fig 10, RQ3, RQ4).

*Please note that the figure numbers have changed due to the restructuring. The figures are now ordered as follows:

Fig 1. Environmental information intervention for treatment groups

Fig 2. Illustration used to test the comprehension of environmental information intervention for treatment groups

Fig 3. An example of DCE question (Japan, Block 1, Question 1)

Fig 4. Behavior and attitude characteristics of the respondents

Fig 5. Respondents’ perceptions toward a DRS for CBs

Fig 6. Simulated adoption rate by cost

Fig 7. WTP monetary comparison

Fig 8. WTP percentage comparison

Fig 9. HNB cigarette usage and familiarity

Fig 10. DRS preferences across tobacco products

2. DCE Design vs. Claims About Feasibility
The introduction and abstract suggest that the study addresses the general feasibility of implementing a DRS for tobacco products. However, the DCE design only captures stated preferences for attributes of a hypothetical scheme. This is not equivalent to assessing real-world feasibility or predicting actual adoption or compliance. The authors should revise claims in the abstract, introduction, and conclusions to focus on preferences rather than feasibility per se.

Our response:

Thank you for pointing this out and providing us with a suggestion. We have added the following sentences in the abstract and introduction to provide further clarification regarding preferences. Concerning the conclusions, we have addressed this point in the subsequent response, as we see it as closely related to the discussion on the limitations of hypothetical scenarios.

Abstract:

[Previous]

This study addresses this gap by examining smokers’ decision-making in hypothetical, real-world DRS scenarios for CBs.

[Revised: LINES 20-21]

This study addresses this gap by investigating smokers’ perceptions and preferences in hypothetical DRS scenarios for CBs.

Introduction:

[Previous]

Despite earlier studies highlighting the potential of a DRS for CBs, two research gaps remain. First, smokers’ perceptions and preferences regarding a DRS for CBs remain underexplored. Adopting new waste collection systems depends on consumer preferences and expected behavior [33]. While Hoek et al. [34] found that 51% of smokers preferred a fixed 2 USD deposit, they did not explore how preferences might vary across different deposit levels. Additionally, although they examined how information about the environmental impact of CB affects perceived responsibility, its influence on the perceptions of a DRS and preferences remains underexplored. Providing such information may increase support for a DRS, aligning with the WHO campaigns to raise awareness of tobacco’s environmental impact [3,4]. Many smokers believe that CBs are biodegradable, a misconception linked to higher littering rates, which also underscores the need for targeted information interventions [35–37]. Second, studies assessing a DRS for CBs across different socioeconomic contexts, particularly between developed and developing countries, are lacking. Developing countries, which often experience higher rates of CB littering, have received limited attention regarding targeted measures [1].

[Revised: LINES 87-102]

Despite earlier studies highlighting the potential of a DRS for CBs, two research gaps remain. First, smokers’ perceptions and preferences regarding a DRS for CBs remain underexplored. Adopting new waste collection systems depends on consumer preferences and expected behavior [34]. While Hoek et al. [35] found that 51% of smokers preferred a fixed 2 USD deposit, they did not explore how preferences might vary across different deposit levels. Additionally, although they examined how information about the environmental impact of CB affects perceived responsibility, its influence on the perceptions of a DRS and preferences remains underexplored. Providing such information may increase support for a DRS, aligning with the WHO campaigns to raise awareness of tobacco’s environmental impact [3,4]. Many smokers believe that CBs are biodegradable, a misconception linked to higher littering rates, which also underscores the need for targeted information interventions [36-38]. This highlights the need for further in-depth research using hypothetical scenarios, enabling the assessment of different attributes, levels, and environmental information to better understand preferences and perceptions that can inform the development of an effective DRS design. Second, studies assessing a DRS for CBs across different socioeconomic contexts, particularly between developed and developing countries, are lacking. Developing countries, which often experience higher rates of CB littering, have received limited attention regarding targeted measures [1].

We also included the following research aim, which emphasizes preferences rather than feasibility.

[LINES 103-104]

To address these gaps, this study investigates smokers’ perceptions of, and preferences for a DRS for CBs, aiming to propose an effective DRS design.

3. Limitations of DCEs in the Tobacco Context
Stated preference methods, such as DCEs, face well-documented challenges in contexts like smoking behaviour, which involve automaticity, time-inconsistent preferences, and attentional limits. These characteristics reduce the external validity of DCEs in predicting actual behaviour.
The discussion and conclusion should more explicitly acknowledge these behavioural and methodological limitations, drawing on the following literature:
• Regmi et al. (2018): A systematic review of DCEs in tobacco control highlights limited behavioural realism and strong influence of monetary attributes in stated preferences.
Regmi, K., Kaphle, D., Timilsina, S., & Tuha, N. A. A. (2018). Application of discrete-choice experiment methods in tobacco control: a systematic review. PharmacoEconomics-Open, 2(1), 5–17.
https://doi.org/10.1007/s41669-017-0030-9
• Quaife et al. (2018): This meta-analysis finds that DCEs have only modest external validity, with an area under the curve (AUC) of 0.68, raising questions about their predictive power for real-world choices.
Quaife, M., Terris-Prestholt, F., Di Tanna, G. L., & Vickerman, P. (2018). How well do discrete choice experiments predict health choices? A systematic review and meta-analysis of external validity. The European Journal of Health Economics, 19(8), 1053–1066.
https://doi.org/10.1007/s10198-018-0960-1
• Haghani et al. (2021): This large-scale review identifies consistent hypothetical bias in stated choice experiments, especially in behavioural domains involving low attention or habitual actions, such as tobacco consumption.
Haghani, M., Bliemer, M. C., Rose, J. M., Oppewal, H., & Lancsar, E. (2021). Hypothetical bias in stated choice experiments: Part I. Macro-scale analysis of literature and integrative synthesis of empirical evidence from applied economics, experimental psychology and neuroimaging. Journal of Choice Modelling, 41, 100309.
https://doi.org/10.1016/j.jocm.2021.100309
This discussion should be used to limit the interpretation of findings and clearly state that the DCE results reflect hypothetical preferences, not observed behaviour or implementation success.

Our response:

Thank you for your suggestion. We appreciate this important point, as indeed, the DCE is hypothetical and may not fully reflect actual preferences or behavior.

In the previous manuscript, we addressed these limitations in the conclusion section. Furthermore, we separated the discussion and conclusion sections; therefore, our conclusion served as the final paragraph, consistent with guidance on writing conclusions (<https://plos.org/resource/how-to-write-conclusions/>), and was structured to highlight limitations and directions for future research. We also reviewed previous papers on structuring such limitations (e.g., Berck et al., 2024; Mutuku et al., 2024).

However, to strengthen this discussion, we have added the suggested references and included the following sentences. In addition, we have incorporated Reviewer #2’s recommendation regarding the design of a specific field experiment:

[Previous]

This study has three limitations. First, because we employed a stated preference methodology, the responses may not fully capture actual preferences. Moreover, the hypothetical scenarios do not consider smokers’ real-world behavior in returning CBs. A field experiment could help validate our findings. Although implementing DRS for CBs may be technically challenging, it is not necessarily impossible given current technologies.

[Revised: LINES 599-607]

This study has three limitations. First, because we employed a stated preference methodology, the responses may not fully capture actual preferences. Moreover, the hypothetical scenarios do not consider smokers’ real-world behavior in returning CBs. The simulated adoption rates by cost were conditional and may not correspond to actual participation rates. Choice experiments are often subject to hypothetical bias and require external validation [109,110]. Thus, a field experiment could help validate our findings. Although implementing DRS for CBs may be technically challenging, it is not necessarily impossible given current technologies. Recreational beaches could serve as ideal sites for such experiments due to their consistently high concentration of CBs and associated metal toxicity leaching [15], as well as the precedent set by behavioral nudge interventions involving smokers [16].

References we used in structuring limitations:

- Berck P, Sears M, Taylor RLC, Trachtman C, Villas-Boas SB. Reduce, reuse, redeem: Deposit-refund recycling programs in the presence of alternatives. Ecol Econ. 2024;217: 108080. doi: [10.1016/j.ecolecon.2023.108080](https://doi.org/10.1016/j.ecolecon.2023.108080).
- Mutuku J, Tocock M, Yanotti M, Tinch D, Hatton MacDonald DH. Public perceptions of the value of reducing marine plastics in Australian waters. Ecol Econ. 2024;217: 108065. doi: [10.1016/j.ecolecon.2023.108065](https://doi.org/10.1016/j.ecolecon.2023.108065).

Recommendations on Tables, Figures, and Presentation
1. Figures 5–7 (WTP and Participation Rates):
o Explicitly explain in the text how WTP values are derived from model coefficients.

Our response:

Thank you for your suggestion. We have added the following equations to explicitly explain in the text how WTP values are derived.

[Previous]

Based on Equation 1, we computed willingness to pay (WTP) by dividing the coefficients of non-monetary attributes (management institution and accessibility) by that of the monetary attribute (cost) [47]. To make the WTP comparable across countries, we computed the percentage of WTP by dividing it by the price of a cigarette pack introduced in DCE choice sets (500 JPY for Japan and 32,000 IDR for Indonesia) [84]. All DCE analyses were conducted using the statistical software R (version 4.3.3) with the mded packages [47].

[Revised: LINES 248-258]

Based on Equation 1, we computed the willingness to pay (WTP) by dividing the coefficients of the non-monetary attributes by the coefficient of the monetary attribute [48]. The equation is given by:

$$WTP=-\frac{\beta_{n}}{\beta_{1}} , n \in\{2, 3\}$$

(3)

To make the WTP comparable across countries, we computed the percentage of WTP by dividing it by the price of a cigarette pack introduced in DCE choice sets (500 JPY for Japan and 32,000 IDR for Indonesia) [85]. All DCE analyses were conducted using the statistical software R (version 4.3.3) with the mded packages [48].

$$WTP \left( \% \right)=\frac{WTP}{Reference price}$$

(4)

o Consider providing a table with means and standard errors for WTP comparisons to support inferences from Figure 6.

Our response:

Thank you for your suggestion. We have added a table in the appendix accordingly.

*Please note that Figure 7 is now the WTP monetary comparison.

*Please note that the appendix numbers have changed due to the addition of a new table. The appendices are now ordered as follows:

S1 Appendix. Eco-guilt items

S2 Appendix. DRS scenario (Japan*)

S3 Appendix. Conditional logit estimates using deposit and refund rate attributes

S4 Appendix. Welch’s test for behavior and attitude characteristics of respondents (Fig 4)

S5 Appendix. WTP means and standard errors (Fig 7)

S6 Appendix. Welch’s test for DRS preferences for tobacco products (Fig 10)

[Revised: LINE 623-626]

S5 Appendix. WTP means and standard errors (Fig 7)

We adopted Krinsky and Robb’s method to calculate 95% confidence intervals [48].

[Revised: Supporting information S5 Appendix]

| WTP management institution | |  |  |  |
| --- | --- | --- | --- | --- |
|  | Japan | | Indonesia | |
|  | Control (n = 911) | Treatment (n = 954) | Control (n = 1,000) | Treatment (n = 1,000) |
| Mean | 16.79 | 47.19 | 404.67 | 631.90 |
| 2.5% quantiles | 6.59 | 25.43 | 135.99 | 325.00 |
| 97.5% quantiles | 29.84 | 102.94 | 710.43 | 1029.30 |
| Standard error | 5.93 | 19.77 | 146.54 | 179.67 |
|  |  |  |  |  |
| WTP accessibility |  |  |  |  |
|  | Japan | | Indonesia | |
|  | Control (n = 911) | Treatment (n = 954) | Control (n = 1,000) | Treatment (n = 1,000) |
| Mean | -5.48 | -9.38 | -84.42 | -151.90 |
| 2.5% quantiles | -8.21 | -20.48 | -126.00 | -222.20 |
| 97.5% quantiles | -3.90 | -5.83 | -53.26 | -108.80 |
| Standard error | 1.10 | 3.74 | 18.56 | 28.93 |

*Please note that the font size in the table has been adjusted to fit within the response brackets. The data are also available in the supporting information, which we kindly ask you to refer to if this response has any size limitations.

Furthermore, we have updated Figures 7 and 8, as there were miscalculations in the Figures in the previous manuscript. The WTP means for management institutions indicate producers’ WTP relative to the grand mean—for example, Japan (control) is 16.79 yen above the grand mean, while the government is 16.79 yen below it (due to effect coding). Thus, the difference between their WTPs is 33.58 yen. The WTP means for accessibility is correct as reported, since Figure 7 is measured on a ratio scale. We have multiplied it by 5, 10, and 30 to reflect the respective attribute levels. We have further clarified this in the appendix caption.

[Further Revised: LINE 623-626]

S5 Appendix. WTP means and standard errors (Fig 7)

We adopted Krinsky and Robb’s method to calculate 95% confidence intervals [48]. The WTP for accessibility appears different in Fig 7 because it has been multiplied to reflect the respective attribute levels.

We are attaching both the original and the revised figures for your reference. The original figures are available in the supporting information, which we kindly ask you to refer to if this response is limited in size.

[Previous: Fig 7]

[Revised: Fig 7]

[Previous: Fig 8]

[Revised: Fig 8]

o Clarify in the figure or caption whether participation rates are conditional or absolute.

Our response:

Thank you for your suggestion. The simulated adoption rate by cost (now Figure 6) are conditional probabilities tied to the logit model structure. This implies that participation probabilities are derived from relative utilities, not absolute preferences.

We have added the following caption to Figure 6 for clarity.

[Previous]

Fig 6. Simulated adoption rate by cost

Opt-out refers to respondents who preferred the status quo (i.e., no DRS).

[Revised: LINE 355-357]

Fig 6. Simulated adoption rate by cost

Opt-out refers to respondents who preferred the status quo (i.e., no DRS). They are conditional probabilities tied to the logit model structure.

2. Table 4 (Conditional Logit Estimates):
o Add statistical significance indicators (e.g., asterisks) for interaction terms.

Our response:

Thank you for your suggestion. We believe that the statistical significance indicators (e.g., asterisks) for the interaction terms are already presented in the Table 4. The meaning of each asterisk is explained in LINE 353-354. However, there are some interaction terms that are not statistically significant and therefore do not have any asterisks. For your reference,

[Table 4]

| ASC:gender |  |  | -0.056 |  |  | 0.302 |
| --- | --- | --- | --- | --- | --- | --- |
|  |  |  | (0.035) |  |  | (0.159) |
| ASC:age |  |  | -0.015*** |  |  | -0.013*** |
|  |  |  | (0.002) |  |  | (0.004) |

Table continues.

*Please note that the font size in the table has been adjusted to fit within the response brackets. The full data are available in Table 4, which we kindly ask you to refer if this response is limited in size.

[LINE 353-354]

***p<0.001; **p<0.01; *p<0.05. Standard errors are shown in parentheses. ASC, alternative-specific constant; AIC, Akaike information criterion; BIC, Bayesian information criterion.

3. Table 1 (Thematic Coding of Perceptions):
o Refer to Table 1 more directly when discussing open-ended perceptions in Section 4.2.

Our response:

Thank you for your suggestion. We see your point; however, we intended Section 4.2 to focus on participants’ overall perceptions of the system (e.g., whether they are supportive of the system or not), rather than their views on the specific attributes or levels presented in the DCE.

Alternatively, we addressed open-ended perceptions of attributes and levels in Section 4.3, Preferences for DRS. Each paragraph focuses on a specific attribute, justifying respondents’ choices and incorporating their open-ended responses. We provide some examples below:

[LINES 446-449]

Respondents in both countries preferred a lower cost for DRSs for CBs (CL 1 model, Table 4). This suggests a reluctance to bear additional financial burdens to support the system’s operation. This may reflect concerns about already high tobacco taxes, and a desire to avoid further costs (No. JP 146, JP 287, ID 296, ID 466).

[LINES 454-456]

Respondents in both countries preferred producer-managed DRS over government-managed DRS (CL 1 model, Table 4). This preference is likely driven by the belief that producers should be responsible for CB waste (No. ID 28, ID 491, ID 501).

[LINES 458-460]

Other reasons cited included the perceived feasibility of producer-managed DRS for CBs (No. ID 434, ID 822), and the desire to minimize the use of public tax funds (JP FG, No. JP 152).

We hope that it is acceptable for you.

o Clarify whether themes map back to RQ2 explicitly.

Our response:

Thank you for your suggestion. We acknowledge that, after inductive coding, the open-ended perceptions of DRS can inform not only RQ2 but also help explain participants’ preferences in relation to RQ3 and RQ4. Consequently, we have incorporated some of the open-ended responses into our discussion of RQ3 and RQ4 (as noted in our previous response). However, the theme map (now Figure 5) was specifically intended to categorize perceptions and explicitly address RQ2. Our aim with the map was to highlight the most prominent themes participants considered when evaluating the system.

We have added a clarification on this, addressing it together with the reviewer’s first comment:

[Revised: LINE 304-305]

Fig 5 illustrates the results of the inductive coding of respondents’ thoughts, ideas, or word associations toward a DRS for CBs (RQ2).

Furthermore, we have added the following sentence at the end of the results section on perceptions of DRS (now Section 3.3) for clarity:

[Revised: LINES 322-323]

We further incorporate respondents’ open-ended responses to discuss preferences for RQ3 and RQ4.

Minor Comments
• Correct the typo in the abstract: "90.33%%" → "90.33%".

Our response:

Thank you for bringing this to our attention. We have corrected it accordingly.

[Previous]

Our results revealed that a DRS for CBs was preferred to the status quo, with higher preferences in Indonesia (90.33%%) than in Japan (63.92%).

[Revised: LINE 25-27]

Our results revealed that a DRS for CBs was preferred to the status quo, with higher preferences in Indonesia (90.33%) than in Japan (63.92%).

• Consider defining “heat-not-burn cigarettes” upon first mention for readers unfamiliar with the term.

Our response:

Thank you for your suggestion. Reviewer #2 also raised this point, and we have accordingly added an explanation of heat-not-burn cigarettes.

[Previous]

Implementation is feasible, as demonstrated in Japan, where collection systems for heat-not-burn (HNB) cigarette components have been established, such as devices, batteries, cartridges, and capsules, owing to fire risks [29–31].

[Revised: LINES 79-83]

Implementation is feasible, as demonstrated in Japan, where collection systems for heat-not-burn (HNB) cigarette components have been established. HNB cigarettes are electronic tobacco products in which the tobacco is heated, rather than combusted, to generate an aerosol. The collection systems cover components such as devices, batteries, cartridges, and capsules, owing to fire safety considerations [30–32].

• Confirm that all country-specific monetary values and exchange rates used in the WTP conversion are properly footnoted or sourced.

Our response:

Thank you for bringing this to our attention. WTP is not calculated using exchange rates; rather, it is derived from the model coefficients of cost (in Japanese Yen and Indonesian Rupiah), which also produces values in JPY and IDR. For reference, however, we have provided a comparison of JPY and IDR to USD in LINE 186-188. We agree that including the source of the conversion is important and have added it accordingly. The date has also been updated to ensure relevance.

[Previous]

JPY, Japanese Yen; IDR, Indonesian Rupiah. 1 JPY = 0.0069 USD, 1 IDR = 0.000061 (June 14, 2025).

[Revised: LINE 186-188]

JPY, Japanese Yen; IDR, Indonesian Rupiah. 1 JPY = 0.0068 USD, 1 IDR = 0.000062 (August 24, 2025, from <https://www.google.com/finance/>).

Reviewer #2: Strengths:

The study addresses a significant environmental issue (cigarette butt pollution) using a novel approach (deposit-refund system, DRS).

The comparative design (Japan vs. Indonesia) provides valuable insights into cultural and socioeconomic differences in smokers' perceptions and preferences.

The use of a discrete choice experiment (DCE) and treatment-control design is methodologically robust and appropriate for assessing hypothetical scenarios.

The inclusion of environmental information intervention is a strong point, as it highlights the role of awareness in shaping behavior.

Dear Reviewer #2,

Thank you for your thoughtful and constructive suggestions. We have carefully revised the manuscript, incorporating many of your suggestions as well as those from Reviewer #1.

After thorough consideration, there are a few suggestions we were unable to adopt upon considering how we can make our manuscript better contribute to the literature. We respectfully hope you understand our reasoning in this regard.

Additionally, please note that Figures 7 (WTP monetary comparison) and 8 (WTP percentage comparison) have been updated to correct miscalculations present in the earlier version. These revisions do not alter our overall findings. We have also updated figure numbers, appendix numbers, and citation numbering throughout the manuscript.

Best regards,

Authors

Areas for Improvement:

Introduction: The introduction could better contextualize the problem of cigarette butt pollution by citing recent studies, such as the comparative study on recreational beaches (referenced below). This would strengthen the justification for the study.

Our response:

Thank you for your suggestion and for drawing our attention to the recommended paper. We have revised the introduction to better contextualize the problem, clarified the impact of CBs, and incorporated the suggested paper to strengthen the framing further.

[Previous]

Littering is a major contributor to environmental pollution [1], with cigarette butts (CBs) being the most littered item, accounting for 4.5 trillion discarded worldwide yearly [2–4]. They were the most collected marine debris during ocean clean-ups, with over 1.8 million retrieved [5]; further, they have been extensively observed in urban areas [6–9]. Cigarette filters are non-biodegradable, and can take up to 14 years to decompose [10–12]. They can release more than 7000 toxic chemicals, including nicotine, heavy metals, polycyclic aromatic hydrocarbons, polycyclic aromatic compounds, and benzene [12–14]. Evidently, these harmful substances leach into soils, plants, and aquatic organisms [6,15–18].

[Revised: LINES 43-53]

Littering is a major contributor to environmental pollution [1], with cigarette butts (CBs) being the most littered item, accounting for 4.5 trillion discarded worldwide yearly [2–4]. Their ubiquity is evident across diverse environments, from urban streets and parks [5-7] to beaches and marine ecosystem, where more than 1.8 million CBs were retrieved during ocean clean-up [8]. The environmental impact of CBs is compounded by their durability, chemical toxicity, and leaching potential [9]. CBs, typically composed of cellulose acetate filters, are not biodegradable under natural conditions and may persist in the environment for up to 14 years [10-12]. Each discarded CB can leach a complex mixture of over 7,000 harmful chemicals, including nicotine, heavy metals (such as cadmium, lead, and arsenic), polycyclic aromatic hydrocarbons, polycyclic aromatic compounds, and benzene [12-15]. These contaminants have been shown to adversely affect soil quality, inhabit plant growth, and pose ingestion risks to aquatic organism [16-21].

Sampling Methods: The manuscript could benefit from a discussion of the limitations of self-reported data (e.g., social desirability bias) and how the inferred valuation method mitigates this.

Our response:

Thank you for your suggestion. We have included the explanation in the discussion section as follows. We did not place it in other sections, such as the conclusion, because self-reported littering was not statistically significant (please kindly refer to Table 4). Thus, firm conclusions about how inferred valuations might mitigate self-reported data could not be drawn. It is also possible that other interaction terms in the model (Equation 2) influenced the results. Additionally, this finding may vary for other types of self-reported measures, as our analysis focused specifically on littering frequencies. Therefore, our explanation was focused on how we related inferred valuations to actual littering behavior.

[LINES 522-531]

Self-reported littering frequencies were insignificant across countries, whereas the inferred valuations of other smokers’ littering frequencies were significant. This discrepancy could be explained by moral considerations and social expectations influencing individuals’ self-reports, leading to underreporting of their own actions [60]. Hence, inferred valuations were considered in relation to actual littering behavior. Interestingly, Japanese respondents with a higher inferred valuation of littering (i.e., littering more) were more likely to prefer DRS for CBs, possibly viewing DRS as a designated disposal point to change their littering habits. In contrast, it was a lower inferred valuation of littering (i.e., littering less) for Indonesian respondents, possibly because they preferred their current littering habits and were less willing to return CBs.

Policy Implications: While the policy implications are well-articulated, they could be expanded to include specific recommendations for integrating DRS with existing waste management systems in each country.

Our response:

Thank you for your suggestion. We have added the following explanation as per your suggestion. Please kindly note that our policy implications are based on the study’s findings regarding smokers’ preferences and perceptions, and are limited to the current waste management systems in each country. That said, we found that the link with the current collection of HNB products could help expand the policy implications.

[Previous]

Fourth, DRS should consider the familiarity and usage of different tobacco products in each country. In regions where HNB cigarettes are widely used, DRS should prioritize their collection alongside CBs. Related studies showed that HNB cigarettes pose environmental toxicity risks [106,107], making their collection crucial for reducing the environmental impact.

[Revised: LINES 566-571]

Fourth, DRS should consider the familiarity and usage of different tobacco products in each country. In regions where HNB cigarettes are widely used, DRS should prioritize their collection alongside CBs. Related studies showed that HNB cigarettes pose environmental toxicity risks [107,108], making their collection crucial for reducing the environmental impact. Existing collection systems, such as those in Japan, use retailers to collect devices [30-32]; these systems could be expanded to include cigarette sticks as well.

Figures and Tables: Ensure all figures and tables are clearly labeled and referenced in the text. Some figures (e.g., Fig 9) lack descriptive captions.

Our response:

Thank you for your suggestion. We recognize that Figure 9 (HNB cigarette usage and familiarity) may appear limited, as its primary purpose is to serve as supporting information. We have made every effort to highlight how Figure 9 illustrates Japanese smokers’ preferences for HNB over CBs by explicitly referring to it in the discussion.

[LINES 384-385]

HNB cigarette users were more prevalent among Japanese than Indonesian respondents, as was familiarity with the products (77.48% and 51.10%, respectively, Fig 9).

[LINES 475-478]

Japanese respondents preferred HNB to CBs, whereas Indonesian respondents preferred CBs to HNB. This difference may primarily stem from Japanese respondents’ greater familiarity with HNB products, with a higher usage rate than that of Indonesian respondents (Fig 9).

Minor Issues:

Typos and grammatical errors (e.g., "as follow-up to 'Financial Disclosure'" appears twice redundantly).

Our response:

Thank you for your observation. It seems that the phrase “as a follow-up to ‘Financial Disclosure’” was not included in our manuscript. Nonetheless, following your suggestion, we have carefully reviewed the manuscript for any typographical or grammatical errors.

The abstract could be more concise; some sentences are overly long.

Our response:

Thank you for bringing this to our attention. We have revised it by adjusting expressions and splitting sentences. We have reduced the words from 260 to 241 words.

[Previous]

Cigarette butts (CBs) are the most littered item worldwide, and contribute significantly to environmental pollution. A deposit-refund system (DRS) has been proposed to reduce CB littering, but its effective design remains underexplored. This study addresses this gap by examining smokers’ decision-making in hypothetical, real-world DRS scenarios for CBs. We conducted a discrete choice experiment in Japan (n = 1,865) and Indonesia (n = 2,000) to investigate their perceptions and preferences. Respondents were divided into treatment and control groups, with the treatment group receiving CB environmental impact information. This information, aligning with WHO’s campaign on tobacco-related environmental harm. Our results revealed that a DRS for CBs was preferred to the status quo, with higher preferences in Indonesia (90.33%%) than in Japan (63.92%). The treatment increased respondents’ preferences for a DRS for CBs (91.82% in Indonesia and 69.83% in Japan), and willingness to endure cost to support DRS operations. Simulated adoption rates varied by cost: in Japan, up to 5% of cigarette price maintained participation probabilities above 55%, while in Indonesia, up to 10% maintained participation probabilities above 80%. Our findings underscore the importance of environmental information in promoting DRS adoption. Both countries preferred a producer-managed system to a government-managed one, highlighting an opportunity for tobacco producers to fulfill extended producer responsibility through a DRS. Furthermore, the DRS design should be adapted to each country’s specific context. Notably, Japanese respondents’ familiarity with heat-not-burn cigarettes influenced their preference for a DRS tailored to those products, whereas Indonesian respondents preferred a DRS for CBs. Japanese respondents also emphasized accessibility more than their Indonesian counterparts.

[Revised: LINES 16-41]

Cigarette butts (CBs) are the world’s most littered item and significantly contribute to environmental pollution. A deposit-refund system (DRS) has been proposed to reduce CB littering, but its effective design remains underexplored. This study addresses this gap by investigating smokers’ perceptions and preferences in hypothetical DRS scenarios for CBs. We conducted a discrete choice experiment in Japan (n = 1,865) and Indonesia (n = 2,000). Respondents were divided into treatment and control groups, with the treatment group receiving information on CB environmental impact aligned with the WHO’s campaign. Our results revealed that a DRS for CBs was preferred to the status quo, with higher preferences in Indonesia (90.33%) than in Japan (63.92%). The information treatment further increased DRS preferences (Indonesia: 91.82%; Japan: 69.83%) and willingness to endure cost to support DRS operations. Cost simulations showed participation probabilities remained above 55% with a cost of up to 5% of a cigarette’s price in Japan, and above 80% with a cost of up to 10% in Indonesia. Our findings underscore the importance of environmental information in DRS adoption. Both countries preferred a producer-managed system to a government-managed one, highlighting an opportunity for tobacco producers to fulfill extended producer responsibility through a DRS. Furthermore, DRS design should be country-specific. Notably, Japanese respondents’ familiarity with heat-not-burn cigarettes influenced their preference for a tailored DRS to those products, whereas Indonesian respondents preferred a DRS for CBs. Japanese respondents also emphasized accessibility more than their Indonesian counterparts.

Suggested Improvements for the Introduction Using the Cited Article

Our response:

Thank you for the suggested improvements. After careful consideration, we have integrated those suggestions that align most closely with the core focus of our manuscript. We respectfully hope that you understand our reasoning in this regard.

The attached article, "Assessing cigarette butt pollution on recreational beaches: A comparative study of two sampling methods and their impact on metal release," provides empirical evidence of cigarette butt pollution's environmental impact, particularly in marine environments. Here’s how you can integrate its findings into your introduction:

Current Problem:

Add: "Cigarette butts are not only the most littered item globally but also a significant source of toxic metal contamination in coastal ecosystems. Recent studies have demonstrated that cigarette butts leach heavy metals like cadmium, lead, and arsenic into beach environments, posing risks to marine life and human health (cite the beach study). This underscores the urgency of addressing cigarette butt pollution beyond urban settings."
*****use this article in manuscript to improve introduction "Assessing cigarette butt pollution on recreational beaches: A comparative study of two sampling methods and their impact on metal release:

Our response:

Thank you for your suggestion. As this point relates closely to the earlier comment on contextualizing the environmental impact of CBs, our response is therefore similar. While we did not adopt the suggested sentences verbatim, we have carefully revised the section to improve clarity and included the recommended citation, taking into account the focus of our manuscript.

Previous]

Littering is a major contributor to environmental pollution [1], with cigarette butts (CBs) being the most littered item, accounting for 4.5 trillion discarded worldwide yearly [2–4]. They were the most collected marine debris during ocean clean-ups, with over 1.8 million retrieved [5]; further, they have been extensively observed in urban areas [6–9]. Cigarette filters are non-biodegradable, and can take up to 14 years to decompose [10–12]. They can release more than 7000 toxic chemicals, including nicotine, heavy metals, polycyclic aromatic hydrocarbons, polycyclic aromatic compounds, and benzene [12–14]. Evidently, these harmful substances leach into soils, plants, and aquatic organisms [6,15–18].

[Revised: LINES 43-53]

Littering is a major contributor to environmental pollution [1], with cigarette butts (CBs) being the most littered item, accounting for 4.5 trillion discarded worldwide yearly [2–4]. Their ubiquity is evident across diverse environments, from urban streets and parks [5-7] to beaches and marine ecosystem, where more than 1.8 million CBs were retrieved during ocean clean-up [8]. The environmental impact of CBs is compounded by their durability, chemical toxicity, and leaching potential [9]. CBs, typically composed of cellulose acetate filters, are not biodegradable under natural conditions and may persist in the environment for up to 14 years [10-12]. Each discarded CB can leach a complex mixture of over 7,000 harmful chemicals, including nicotine, heavy metals (such as cadmium, lead, and arsenic), polycyclic aromatic hydrocarbons, polycyclic aromatic compounds, and benzene [12-15]. These contaminants have been shown to adversely affect soil quality, inhabit plant growth, and pose ingestion risks to aquatic organism [16-21].

Gaps in Existing Measures:

Expand: "While measures like fines and clean-up initiatives target urban littering, they are less effective in recreational areas such as beaches, where cigarette butts are often buried in sand and overlooked by traditional sampling methods (cite the beach study). This highlights the need for systemic solutions like DRS, which can incentivize proper disposal across diverse environments."

Our response:

Thank you for your suggestion. We see your point; however, we would like to keep this section succinct, providing an overview without going into extensive detail (e.g., we aimed to avoid focusing too narrowly on specific environments such as beaches). We also acknowledge that the gap in existing measures is largely attributable to the small size of CBs (as the reviewer also noted, they are often buried and overlooked), which we have addressed in the following section.

[LINES 64-66]

Challenges stem from CBs’ small size, low economic value, and ingrained disposal habits [9,13,25]. Tossing CBs is often a habitual part of smoking, and is widely considered acceptable [13].

Regarding the point that “DRS can incentivize proper disposal across diverse environments,” we also recognize that other measures (e.g., taxes, fines, clean-up) are designed to apply across different settings, not only urban areas. For this reason, we were careful in distinguishing how DRS differs from these measures, which we have addressed below.

[LINES 68-76]

Recently, a deposit-refund system (DRS) has been proposed as a promising solution to address CB littering [9,12,27,28]. Under this system, smokers pay a deposit included in the cigarette price, refunded upon returning CBs. Two key features make the DRS promising: financial incentives and support for recycling or upcycling. First, the refund offers financial incentives to motivate returns [9], reduces litter and cleanup costs, and creates income opportunities for informal waste collectors. Second, the DRS can support CB recycling or upcycling, currently incinerated or landfilled, both of which are unsuitable [25], into products such as bricks, roads, energy sources, or insecticides [12,29]. Therefore, the DRS addresses current waste management gaps by offering safe disposal options [25].

Justification for DRS:

Link: "The persistence of cigarette butts in marine environments, as evidenced by their high metal leaching potential (cite the beach study), aligns with our findings on smokers' misconceptions about biodegradability. A DRS could mitigate this by ensuring proper collection and recycling, reducing environmental toxicity."

Our response:

Thank you for your suggestion. We are concerned that our findings on misconceptions about biodegradability may not fit naturally into the introduction. Our intention was to use these findings to highlight smokers’ knowledge and the need for environmental information, rather than to explain the persistence of CBs in marine settings. Regarding the justification for a DRS to ensure proper collection, we have added the following paragraph, which we believe adequately addresses the reviewer’s point and aligns with the last proposed sentence.

[LINES 70-76]

Two key features make the DRS promising: financial incentives and support for recycling or upcycling. First, the refund offers financial incentives to motivate returns [9], reduces litter and cleanup costs, and creates income opportunities for informal waste collectors. Second, the DRS can support CB recycling or upcycling, currently incinerated or landfilled, both of which are unsuitable [25], into products such as bricks, roads, energy sources, or insecticides [12,29]. Therefore, the DRS addresses current waste management gaps by offering safe disposal options [25].

Comparative Context:

Emphasize: "The beach study's comparison of sampling methods also mirrors our cross-country approach, revealing how context-specific factors (e.g., beach tourism in Indonesia vs. urban littering in Japan) influence pollution patterns. Tailoring DRS designs to these differences is critical for success."

Our response:

Thank you for your suggestion. While our cross-country approach indeed highlights the need for context-specific DRS design, our contribution is primarily focused on smokers’ preferences and perceptions of the system, rather than on pollution patterns.

That said, we agree with the importance of comparative context in illustrating how studies may differ or align with one another. We have addressed this by highlighting how our findings relate to previous work.

[LINES 402-404]

Japanese and Indonesian respondents in our study demonstrated a better understanding of the environmental impact of CBs than those in previous studies [35,37,90], except for non-biodegradability (Table 3).

[LINES 440-442]

This suggests that respondents recognized the effectiveness of DRS in reducing CB littering, with their preferences exceeding 51% of smokers in New Zealand, who viewed cigarette deposits as an effective anti-littering measure [35].

[LINES 466-468]

Both Japanese and Indonesian respondents emphasized DRS accessibility (CL 1 model, Table 4). This aligns with consumers’ tendency to prefer the nearest facility when travel is required [93], and mirrors the findings on beverage containers DRS [94,95].

Additional Recommendations

Clarify Terminology: Define "heat-not-burn (HNB) cigarettes" early in the introduction, as not all readers may be familiar with this product.

Our response:

Thank you for your suggestion. Reviewer #1 also raised this point, and we have accordingly added an explanation of heat-not-burn cigarettes.

[Previous]

Implementation is feasible, as demonstrated in Japan, where collection systems for heat-not-burn (HNB) cigarette components have been established, such as devices, batteries, cartridges, and capsules, owing to fire risks [29–31].

[Revised: LINES 79-83]

Implementation is feasible, as demonstrated in Japan, where collection systems for heat-not-burn (HNB) cigarette components have been established. HNB cigarettes are electronic tobacco products in which the tobacco is heated, rather than combusted, to generate an aerosol. The collection systems cover components such as devices, batteries, cartridges, and capsules, owing to fire safety considerations [30–32].

Highlight Innovation: Stress how your study advances prior work (e.g., by testing cost thresholds and producer-managed systems, which are underexplored in the literature).

Our response:

Thank you for your suggestion. We have added the following explanation as per your suggestion.

[Previous]
This study is the first to examine smokers’ perceptions and preferences regarding a DRS for CBs. Using a stated preference DCE, we explored a range of scenarios, attributes, levels, and information interventions that would be difficult to replicate in a real-world setting, thereby contributing to the literature on effective DRS design. Our study reveals the importance of how smokers perceive deposit and refund rate as a combined attribute, which we defined as cost, an approach that can inform and benefit future DRS research.

[Revised: LINES 580-587]

This study is the first to examine smokers’ perceptions and preferences regarding a DRS for CBs. Using a stated preference DCE, we explored a range of scenarios, attributes, levels, and information interventions that would be difficult to replicate in a real-world setting, thereby contributing to the literature on effective DRS design. Our study reveals the importance of how smokers perceive deposit and refund rate as a combined attribute, which we defined as cost, an approach that can inform and benefit future DRS research. Additionally, we attempted to simulate adoption rates based on this cost measure, extending prior research by offering insights into how varying cost levels may influence participation probabilities and inform policy decisions aimed at maximizing uptake.

Future Research: Suggest field experiments to validate DCE results, citing the beach study's methodological rigor as a model.

Our response:

Thank you for your suggestion. In the conclusion, we proposed a field experiment to validate the DCE results. Upon further reflection, we recognize the value of discussing how such an experiment could be conducted, as suggested with reference to beaches. We have also incorporated Reviewer #1’s comment regarding the limitations of the DCE.

[Previous]

This study has three limitations. First, because we employed a stated preference methodology, the responses may not fully capture actual preferences. Moreover, the hypothetical scenarios do not consider smokers’ real-world behavior in returning CBs. A field experiment could help validate our findings. Although implementing DRS for CBs may be technically challenging, it is not necessarily impossible given current technologies.

[Revised: LINES 599-607]

This study has three limitations. First, because we employed a stated preference methodology, the responses may not fully capture actual preferences. Moreover, the hypothetical scenarios do not consider smokers’ real-world behavior in returning CBs. The simulated adoption rates by cost were conditional and may not correspond to actual participation rates. Choice experiments are often subject to hypothetical bias and require external validation [109,110]. Thus, a field experiment could help validate our findings. Although implementing DRS for CBs may be technically challenging, it is not necessarily impossible given current technologies. Recreational beaches could serve as ideal sites for such experiments due to their consistently high concentration of CBs and associated metal toxicity leaching [15], as well as the precedent set by behavioral nudge interventions involving smokers [16].
